# Association of the HALP score with baseline and incident physical-cognitive comorbidity and mortality: evidence from the WCHAT and UK Biobank cohorts

**DOI:** 10.3389/fpubh.2026.1842125

**Published:** 2026-05-22

**Authors:** Chaoxin Gao, Yiheng Zhou, Rongna Lian, Birong Dong, Jirong Yue, Haiyang Chen, Xiaolei Liu

**Affiliations:** 1 Department of Geriatrics and National Clinical Research Center for Geriatrics, Laboratory of Metabolism and Aging, West China Hospital, Sichuan University, Chengdu, China; 2 General Practice Ward/International Medical Center Ward, General Practice Medical Center, West China Hospital, Sichuan University, Chengdu, China

**Keywords:** cohort study, HALP score, immune-nutritional status, incidence, mortality, physical-cognitive comorbidity

## Abstract

**Objective:**

To evaluate the associations of the hemoglobin-albumin-lymphocyte-platelet (HALP) score with baseline physical-cognitive comorbidity, incident physical-cognitive comorbidity, and all-cause mortality using two independent cohorts.

**Methods:**

Data were derived from the West China Health and Aging Trend (WCHAT) cohort and the UK Biobank (UKB). Participants aged ≥50 years with available laboratory, covariate, and functional data were included. Physical-cognitive comorbidity was defined as the coexistence of low grip strength and cognitive impairment. Multivariable logistic regression was used for cross-sectional analyses in both cohorts. Cox proportional hazards models were used to examine incident physical-cognitive comorbidity in WCHAT and all-cause mortality among participants with baseline comorbidity in UKB. Continuous-variable, subgroup, sensitivity, and restricted cubic spline analyses were additionally performed, and an exploratory incidence analysis was conducted in UKB participants without baseline comorbidity.

**Results:**

A total of 5,957 participants in WCHAT and 101,655 participants in UKB were included in the baseline analyses. In both cohorts, higher HALP scores were associated with a lower risk of baseline physical-cognitive comorbidity. In the fully adjusted models, the odds ratio comparing Quartile 4 (Q4) with Quartile 1 (Q1) was 0.79 (95% CI: 0.62–0.99) in WCHAT and 0.77 (95% CI: 0.66–0.89) in UKB. In WCHAT, among 2,782 participants free of comorbidity at baseline, 330 incident events occurred, and higher HALP was associated with lower incident risk (Q4 vs. Q1: HR = 0.70, 95% CI: 0.52–0.94; per 1-SD increase: HR = 0.88, 95% CI: 0.79–0.99). In UKB, among 1,393 participants with baseline comorbidity, 227 deaths occurred, and higher HALP was associated with lower all-cause mortality (Q4 vs. Q1: HR = 0.65, 95% CI: 0.45–0.93; per 1-SD increase: HR = 0.80, 95% CI: 0.70–0.92). Findings were generally consistent in subgroup and sensitivity analyses, whereas time-dependent ROC-AUC analysis showed limited discrimination of the HALP-only model.

**Conclusion:**

Higher HALP scores were associated with lower risks of baseline and incident physical-cognitive comorbidity and with better survival among participants with baseline physical-cognitive comorbidity. HALP may provide complementary information for immune-nutritional risk assessment, but it should not be interpreted as an independent predictive tool.

## Introduction

With the continued acceleration of global population aging, the health burden arising from the coexistence of physical functional decline and cognitive impairment has become a major concern in geriatrics and public health (1). In recent years, concepts such as “cognitive frailty” have been used to describe this complex geriatric syndrome, emphasizing the coexistence and clinical relevance of physical vulnerability and cognitive impairment (2). Systematic reviews and meta-analyses have shown that middle-aged and older adults with concurrent physical dysfunction and cognitive impairment are more likely to experience disability, hospitalization, dementia, and all-cause mortality than those with only one functional abnormality (3, 4). Therefore, identifying simple indicators that can detect high-risk individuals before or during the development of physical-cognitive comorbidity is of important clinical and public health value.

Among indicators of physical function, grip strength is widely regarded as an important surrogate for overall muscle strength and physiological reserve because it is easy to measure, inexpensive, and highly reproducible (5). Previous prospective studies and meta-analyses have shown that lower grip strength is closely associated with increased risks of cognitive impairment, dementia, and Alzheimer’s disease (6). A pooled longitudinal analysis across multiple cohorts further showed that higher grip strength was associated with a slower rate of cognitive decline and a lower risk of cognitive impairment, suggesting that physical functional decline and cognitive deterioration may evolve in parallel (7). In addition, studies in community-dwelling middle-aged and older adults have shown that baseline physical frailty predicts subsequent cognitive decline (8). In Chinese middle-aged and older adults, the West China Health and Aging Trend (WCHAT) study also indicated that physical frailty is closely related to cognitive impairment and exhibits distinct co-occurrence patterns and influencing factors (9). Together, these findings suggest that physical functional decline and cognitive impairment should be studied not as two isolated problems, but as an integrated geriatric syndrome.

From a mechanistic perspective, anemia, malnutrition, chronic low-grade inflammation, and immune imbalance are considered key shared pathways linking physical functional decline and cognitive impairment (10). Previous systematic reviews and meta-analyses have shown that anemia is significantly associated with cognitive decline and increased dementia risk, possibly through insufficient oxygen delivery, abnormal cerebral perfusion, and related pathological processes (11, 12). Low albumin levels not only indicate inadequate nutritional reserve but are also regarded as biological markers of systemic frailty and poor prognosis, and they have been associated with cognitive decline and adverse outcomes in middle-aged and older adults (13). Meanwhile, persistent low-grade inflammation and immune dysregulation are also thought to jointly promote muscle weakness, physical frailty, and neurocognitive decline (14). Therefore, compared with single indicators, a composite index integrating hematologic, nutritional, inflammatory, and immune information may better identify middle-aged and older adults at high risk for physical-cognitive comorbidity.

The HALP score comprises hemoglobin, albumin, lymphocytes, and platelets, and it was originally proposed as a prognostic index for patients with gastric cancer (15). Biologically, hemoglobin and albumin reflect oxygen-carrying capacity and nutritional reserve, respectively, whereas lymphocytes and platelets may, to some extent, reflect immune status, inflammatory activation, and thrombo-inflammatory responses (16, 17). In recent years, the application of HALP has gradually expanded to non-neoplastic diseases. Previous studies have shown that a lower HALP score is associated with an increased risk of post-stroke cognitive impairment, suggesting its potential value for risk stratification in neurological disorders as well (18).

Against this background, we used two independent large-scale cohorts, the West China Health and Aging Trend (WCHAT) study and the UK Biobank (UKB), to examine the associations of the HALP score with physical-cognitive comorbidity and its prognosis (19, 20). The WCHAT study is a prospective cohort of community-dwelling middle-aged and older adults in western China, with relatively complete baseline and follow-up assessments. The UK Biobank is a large population-based prospective cohort in the United Kingdom, with a large sample size and long-term follow-up. In the present study, WCHAT was mainly used to evaluate incident physical-cognitive comorbidity, whereas UK Biobank was mainly used to evaluate all-cause mortality among participants with baseline physical-cognitive comorbidity and to provide a supplementary exploratory incidence analysis. We hypothesized that a higher HALP score, as a composite marker of better immune-nutritional status, would be independently associated with lower risk of physical-cognitive comorbidity and better survival.

## Materials and methods

### Study design and population

**Figure 1:**
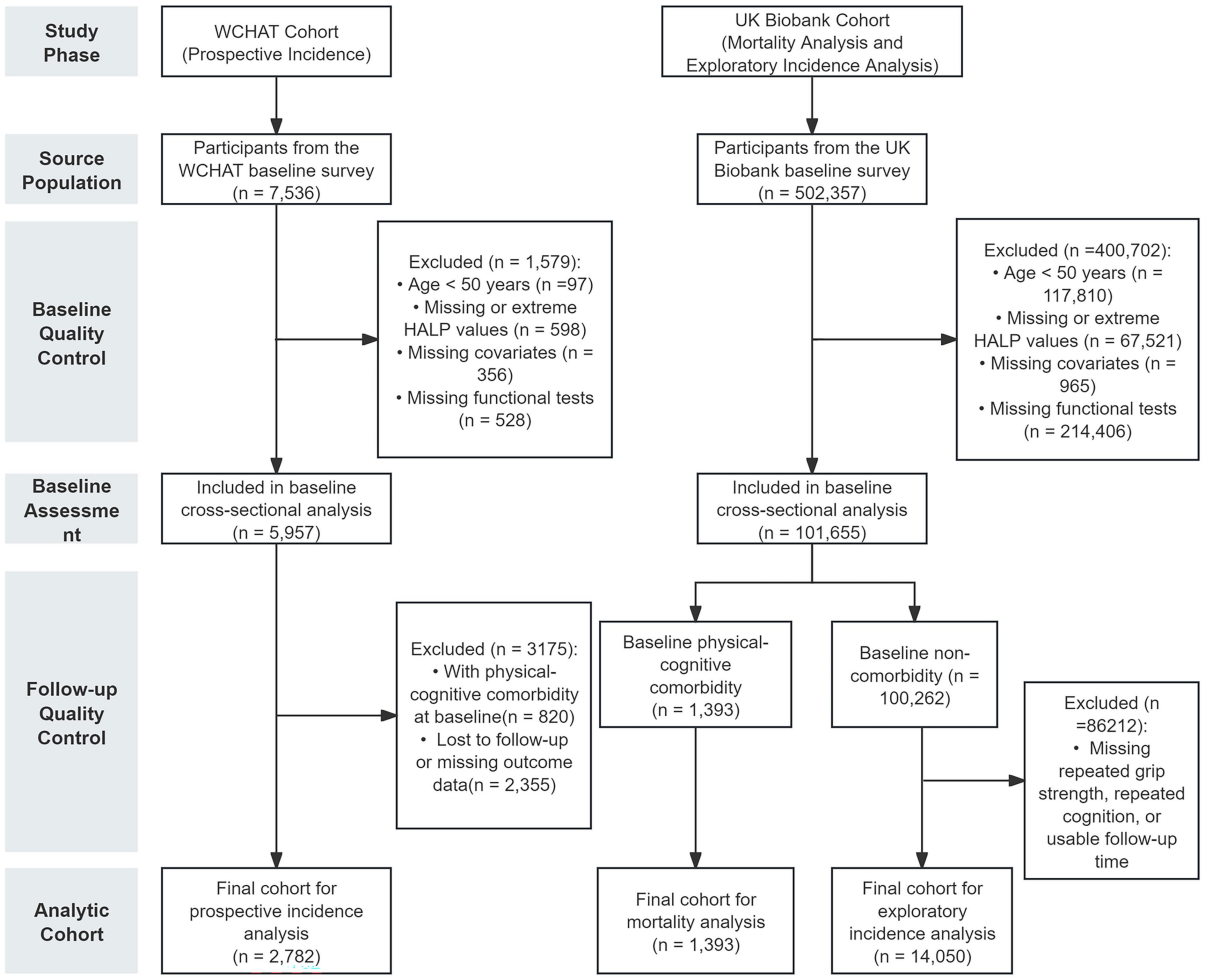
Study design and flowchart of participant selection in the WCHAT and UK Biobank cohorts. This flowchart illustrates the multi-stage recruitment and exclusion process for the WCHAT cohort used for prospective incidence analysis, and for the UK Biobank cohort used for baseline cross-sectional analysis, mortality analysis among participants with baseline physical-cognitive comorbidity, and exploratory incidence analysis among participants without baseline comorbidity.

This study was based on two independent population-based cohorts: the West China Health and Aging Trend (WCHAT) study and the UK Biobank (UKB) (19, 20). The WCHAT study is a prospective cohort of community-dwelling middle-aged and older adults in western China. At baseline in 2018, the cohort enrolled 7,536 participants, including 7,439 individuals aged 50 years and older, and collected relatively complete information on demographic characteristics, lifestyle factors, laboratory indicators, physical function, and cognitive status, with repeated follow-up assessments. The UK Biobank is a large-scale population-based prospective cohort in the United Kingdom. Between 2006 and 2010, more than 500,000 participants aged 40–69 years were recruited from across England, Wales, and Scotland, with extensive baseline information and long-term follow-up for major health outcomes. The participant selection flowchart is shown in Figure 1.

This study was conducted in accordance with the Declaration of Helsinki. The WCHAT protocol was approved by the Biomedical Ethics Review Committee of West China Hospital, Sichuan University, and the UK Biobank study was approved by the North West Multi-center Research Ethics Committee. Written informed consent was obtained from all participants prior to enrollment.

Among WCHAT participants, the present study focused on middle-aged and older adults; therefore, age ≥50 years was used as the primary inclusion threshold in order to capture an earlier stage at which physical functional decline and cognitive deterioration may begin to accumulate, while also maintaining consistency across the two cohorts. After excluding participants with missing or extreme HALP values, missing key covariates, or missing baseline functional assessments, 5,957 individuals were included in the baseline cross-sectional analysis. After further excluding those with baseline physical-cognitive comorbidity and those lost to follow-up or with indeterminate outcomes, 2,782 participants remained for the prospective incidence analysis.

In the UK Biobank cohort, the original sample consisted of 502,357 participants. After applying the age criterion (≥50 years), HALP quality control, and screening for covariates and baseline functional data, 101,655 participants were included in the baseline cross-sectional analysis. Among them, 1,393 participants with baseline physical-cognitive comorbidity were included in the all-cause mortality analysis, and 14,050 participants without baseline comorbidity but with available repeated grip strength, repeated cognitive assessments, and valid follow-up time were included in the exploratory incidence analysis.

### Exposure, covariates, and outcome definitions

The primary exposure was the HALP score, which was used to characterize immune-nutritional status. Based on fasting venous blood measurements, HALP was calculated as follows:

HALP=(hemoglobin[g/L]×10×albumin[g/L]×lymphocytes[10^9/L])/platelets[10^9/L].

In the primary analyses, HALP was categorized into quartiles (Q1–Q4), with the lowest quartile (Q1) serving as the reference group. In supplementary analyses, HALP was entered into the models as a continuous variable and standardized as a Z score, with effect estimates reported per 1-standard deviation (SD) increase. To reduce the influence of outliers, extreme HALP values were defined according to cohort-specific distributions as observations below the 1st percentile or above the 99th percentile.

Physical-cognitive comorbidity was defined as the coexistence of low grip strength and cognitive impairment. In the WCHAT cohort, low grip strength was defined according to the Asian Working Group for Sarcopenia (AWGS 2019) criteria as <28.0 kg in men and <18.0 kg in women (21), and cognitive impairment was assessed using the Short Portable Mental Status Questionnaire (SPMSQ), with a score >2 indicating cognitive impairment (22). In the UK Biobank cohort, low grip strength was defined according to the European Working Group on Sarcopenia in Older People 2 (EWGSOP2) criteria as <27.0 kg in men and <16.0 kg in women (23). Cognitive impairment was based on an operational definition used in previous UK Biobank studies and was defined as a fluid intelligence score more than 1 SD below the mean of the baseline analytical sample (24). In both WCHAT and UK Biobank, maximum grip strength from both hands was used as the analytic grip strength variable.

This study included three levels of analysis. First, both WCHAT and UK Biobank were used for baseline cross-sectional analyses to evaluate the association between HALP and baseline physical-cognitive comorbidity. Second, WCHAT was used to evaluate the association between HALP and incident physical-cognitive comorbidity. Third, UK Biobank participants with baseline comorbidity were used to evaluate the association between HALP and all-cause mortality. In addition, an exploratory incidence analysis was conducted among UK Biobank participants without baseline comorbidity.

### Covariate adjustment strategy

The same sequential covariate adjustment strategy was applied to both the logistic regression analyses for baseline physical-cognitive comorbidity and the Cox proportional hazards regression analyses for longitudinal outcomes. Model 1 was unadjusted. Model 2 was adjusted for age and sex. Model 3 was the fully adjusted model, including age, body mass index, sex, education, race/ethnicity, smoking status, alcohol drinking status, hypertension, diabetes, and coronary heart disease. For the WCHAT incidence analysis, baseline single impairment status, defined as the presence of either low grip strength alone or cognitive impairment alone at baseline, was additionally included in Model 3 to account for pre-existing functional or cognitive vulnerability before the onset of physical-cognitive comorbidity.

### Statistical analysis

All statistical analyses were performed using Python (version 3.12). Continuous variables are presented as means ± standard deviations, and categorical variables as counts (percentages). For baseline comparisons, continuous variables were analyzed using Student’s *t*-test with Welch’s correction, and categorical variables using the χ^2^ test.

Multivariable logistic regression was used to assess the association between HALP and baseline physical-cognitive comorbidity, with results reported as odds ratios (ORs) and 95% confidence intervals (CIs). Cox proportional hazards models were used for incident physical-cognitive comorbidity in WCHAT and all-cause mortality in UK Biobank, with results reported as hazard ratios (HRs) and 95% CIs. In the UK Biobank mortality analysis, Kaplan–Meier survival curves were plotted and compared across HALP quartiles using the log-rank test; pairwise *post hoc* comparisons between quartiles were further adjusted using the Bonferroni method.

In addition to quartile-based analyses, HALP was also modeled as a continuous variable, and the associations with the outcomes were estimated per 1-SD increase. To explore potential nonlinear dose–response relationships, restricted cubic spline (RCS) models with four knots were fitted on the basis of the fully adjusted Cox models (25). Considering the right-skewed distribution of HALP, HALP was first natural-log transformed to reduce skewness and the influence of extreme high values (26), and the log-transformed HALP was then standardized before RCS modeling. The RCS plots were presented on the same transformed scale used for model fitting, namely the Z-score of ln (HALP). For each RCS analysis, both the *p*-value for the overall association and the p value for non-linearity were evaluated.

To evaluate the consistency of the findings across different populations, subgroup analyses were conducted and multiplicative interactions between HALP and stratification variables were tested. Stratification variables included age, sex, BMI, smoking, alcohol drinking, and chronic disease status. Two types of sensitivity analyses were also performed: first, analyses excluding events that occurred within the first year of follow-up to reduce potential reverse causation; and second, analyses restricted to participants aged ≥60 years to evaluate the robustness of the age threshold used in the main analyses.

In addition, we compared baseline characteristics between participants included in and excluded from the prospective analyses and conducted an exploratory incidence analysis among UK Biobank participants without baseline comorbidity. To evaluate the predictive performance boundary of HALP as a single indicator, HALP-only Cox proportional hazards models were fitted, and model discrimination was evaluated using Harrell’s C-index and time-dependent receiver operating characteristic curve analysis. Because the log-transformed and standardized RCS analyses did not show statistically significant nonlinearity or visually obvious turning points, exploratory threshold effect analyses were not further performed. A two-sided *p* < 0.05 was considered statistically significant.

## Results

### Participant selection and baseline characteristics

The participant selection flowchart is shown in Figure 1. In WCHAT, 7,536 baseline participants were initially considered. After excluding those aged <50 years and those with missing or extreme HALP values, missing covariates, or missing functional assessments, 5,957 participants were included in the baseline cross-sectional analysis. After further excluding participants with baseline physical-cognitive comorbidity and those lost to follow-up or with missing outcome data, 2,782 participants were ultimately included in the prospective incidence analysis. In UK Biobank, 502,357 baseline participants were initially screened. After the same quality-control procedures, 101,655 participants were included in the baseline cross-sectional analysis; among them, 1,393 participants with baseline physical-cognitive comorbidity entered the mortality analysis, and 14,050 participants without baseline comorbidity entered the supplementary exploratory incidence analysis. Variable-level missingness, the definition of extreme HALP values, and the stepwise exclusion process are shown in Supplementary Table S1; comparisons between included and excluded participants in the prospective analyses are shown in Supplementary Table S2.

Baseline characteristics of the two cohorts are presented in Table 1. In WCHAT, compared with the non-comorbidity group, the comorbidity group was older, had a lower BMI, lower HALP scores, worse cognitive scores, lower grip strength, and higher proportions of women, participants with lower education, and participants from minority ethnic groups. In UK Biobank, the comorbidity group was likewise older, had poorer cognitive performance and lower grip strength, and had higher proportions of hypertension, diabetes, and coronary heart disease. In both cohorts, HALP scores were lower in the comorbidity group than in the non-comorbidity group, although this difference did not reach statistical significance in UK Biobank.

**Table 1:** Baseline characteristics of participants in the WCHAT and UK Biobank cohorts according to baseline physical-cognitive comorbidity status.

Variables	WCHAT (*N* = 5,957)			UK Biobank (*N* = 101,655)		
	Non-comorbidity (*n* = 5,137)	Comorbidity (*n* = 820)	*P*-value	Non-comorbidity (*n* = 100,262)	Comorbidity (*n* = 1,393)	*P*-value
Age (years)	61.62 ± 7.94	66.79 ± 8.91	<0.001	60.22 ± 5.40	61.73 ± 5.44	<0.001
BMI (kg/m^2^)	25.57 ± 4.00	24.49 ± 4.58	<0.001	27.47 ± 4.71	28.47 ± 5.50	<0.001
HALP score	73.50 ± 30.92	66.25 ± 31.06	<0.001	54.67 ± 19.54	54.39 ± 21.27	0.624
Cognitive score^a^	0.89 ± 1.30	4.35 ± 1.43	<0.001	6.05 ± 2.11	2.45 ± 0.73	<0.001
Grip strength (kg)	23.53 ± 8.72	14.15 ± 4.59	<0.001	31.14 ± 10.70	16.08 ± 6.09	<0.001
Sex, n (%)			<0.001			0.005
Female	3,123 (60.8%)	622 (75.9%)		53,968 (53.8%)	803 (57.6%)	
Male	2014 (39.2%)	198 (24.1%)		46,294 (46.2%)	590 (42.4%)	
Education, n (%)			<0.001			<0.001
Below junior high school	2,891 (56.3%)	763 (93.0%)		30,575 (30.5%)	889 (63.8%)	
Junior high school and above	2,246 (43.7%)	57 (7.0%)		69,687 (69.5%)	504 (36.2%)	
Race, n (%)			<0.001			<0.001
Recognized minority group	3,049 (59.4%)	582 (71.0%)		11,643 (11.6%)	467 (33.5%)	
Majority/dominant group	2088 (40.6%)	238 (29.0%)		88,619 (88.4%)	926 (66.5%)	
Smoking, n (%)			<0.001			<0.001
No	4,126 (80.3%)	712 (86.8%)		91,675 (91.4%)	1,217 (87.4%)	
Yes	1,011 (19.7%)	108 (13.2%)		8,587 (8.6%)	176 (12.6%)	
Alcohol use, n (%)			<0.001			<0.001
No	4,112 (80.0%)	718 (87.6%)		7,694 (7.7%)	323 (23.2%)	
Yes	1,025 (20.0%)	102 (12.4%)		92,568 (92.3%)	1,070 (76.8%)	
Hypertension, n (%)			0.577			<0.001
No	2,317 (45.1%)	379 (46.2%)		69,530 (69.3%)	814 (58.4%)	
Yes	2,820 (54.9%)	441 (53.8%)		30,732 (30.7%)	579 (41.6%)	
Diabetes, n (%)			0.016			<0.001
No	4,410 (85.8%)	730 (89.0%)		94,288 (94.0%)	1,172 (84.1%)	
Yes	727 (14.2%)	90 (11.0%)		5,974 (6.0%)	221 (15.9%)	
Coronary heart disease, n (%)			0.123			<0.001
No	4,739 (92.3%)	743 (90.6%)		95,193 (94.9%)	1,223 (87.8%)	
Yes	398 (7.7%)	77 (9.4%)		5,069 (5.1%)	170 (12.2%)	

Continuous variables are presented as mean ± standard deviation (SD), and categorical variables are presented as number (percentage). ^a^Cognitive score indicates the SPMSQ score in the WCHAT cohort (higher scores indicate worse cognitive function) and the Fluid Intelligence score in the UK Biobank cohort (lower scores indicate worse cognitive function). Grip strength indicates maximum grip strength derived from both hands in both the WCHAT cohort and the UK Biobank cohort. BMI, body mass index; HALP, hemoglobin-albumin-lymphocyte-platelet score.

To quantify heterogeneity in the operational definitions of physical-cognitive comorbidity across cohorts, we further summarized the baseline prevalence and follow-up event rates under the cohort-specific definitions used in WCHAT and UK Biobank (Supplementary Table S3). The results showed clear differences between WCHAT and UK Biobank in the definitions of low grip strength and cognitive impairment, indicating that the identified comorbidity phenotypes were not entirely equivalent in severity. These findings support focusing on the direction of association, robustness, and biological consistency rather than mechanically comparing absolute effect sizes across the two cohorts.

### Cross-sectional associations between HALP and baseline physical-cognitive comorbidity

Cross-sectional results are shown in Table 2. In WCHAT, using the lowest HALP quartile (Q1) as the reference, both the unadjusted and minimally adjusted models showed that higher HALP quartiles were associated with lower risk of baseline physical-cognitive comorbidity. In the fully adjusted model, the association was attenuated but remained significant: compared with Q1, the ORs for Q3 and Q4 were 0.75 (95% CI: 0.60–0.94) and 0.79 (95% CI: 0.62–0.99), respectively. In UK Biobank, after full adjustment, the ORs for Q2, Q3, and Q4 versus Q1 were 0.83 (95% CI: 0.71–0.96), 0.72 (95% CI: 0.62–0.84), and 0.77 (95% CI: 0.66–0.89), respectively, indicating that higher HALP scores were independently associated with lower risk of baseline physical-cognitive comorbidity.

**Table 2:** Cross-sectional associations between HALP score and baseline physical-cognitive comorbidity in the WCHAT and UK Biobank cohorts.

Model	HALP quartiles	WCHAT cohort OR (95% CI)	*P*-value	UK Biobank cohort OR (95% CI)	*P*-value
Model 1	Q1 (Ref)	1	–	1	–
	Q2	0.72 (0.59, 0.87)	<0.001	0.83 (0.72, 0.96)	0.012
	Q3	0.56 (0.46, 0.69)	<0.001	0.76 (0.65, 0.88)	<0.001
	Q4	0.52 (0.42, 0.64)	<0.001	0.92 (0.80, 1.07)	0.274
Model 2	Q1 (Ref)	1	–	1	–
	Q2	0.77 (0.63, 0.94)	0.011	0.84 (0.72, 0.97)	0.018
	Q3	0.62 (0.50, 0.76)	<0.001	0.78 (0.67, 0.90)	0.001
	Q4	0.59 (0.47, 0.73)	<0.001	0.96 (0.83, 1.10)	0.535
Model 3	Q1 (Ref)	1	–	1	–
	Q2	0.88 (0.71, 1.09)	0.238	0.83 (0.71, 0.96)	0.014
	Q3	0.75 (0.60, 0.94)	0.011	0.72 (0.62, 0.84)	<0.001
	Q4	0.79 (0.62, 0.99)	0.04	0.77 (0.66, 0.89)	<0.001

Results are presented as odds ratios (ORs) and 95% confidence intervals (CIs). Model 1 was unadjusted. Model 2 was adjusted for age and sex. Model 3 was fully adjusted for age, BMI, sex, education, race/ethnicity, smoking, alcohol use, hypertension, diabetes, and coronary heart disease (CHD). HALP, hemoglobin-albumin-lymphocyte-platelet score.

When HALP was analyzed as a continuous variable, the results were generally consistent with the quartile analyses (Supplementary Table S4). In the fully adjusted model, each 1-SD increase in HALP was associated with a 9% lower risk of baseline comorbidity in WCHAT (OR = 0.91, 95% CI: 0.84–0.99) and an 8% lower risk in UK Biobank (OR = 0.92, 95% CI: 0.87–0.97). Cross-sectional subgroup analyses are presented in Supplementary Table S5. No significant interactions were observed in WCHAT. In UK Biobank, statistically significant interactions were observed in strata defined by education, BMI, and hypertension, whereas the direction of association was generally consistent across the remaining strata.

### Prospective associations between HALP and incident physical-cognitive comorbidity in WCHAT

In WCHAT, 2,782 participants free of physical-cognitive comorbidity at baseline were included in the prospective analysis, during which 330 incident events occurred over a median follow-up of 5 years (IQR: 3–5 years). The Cox proportional hazards results are shown in Table 3. After full adjustment, compared with Q1, the risks of incident physical-cognitive comorbidity were reduced by 40, 31, and 30% in Q2, Q3, and Q4, respectively, corresponding to HRs of 0.60 (95% CI: 0.44–0.81), 0.69 (95% CI: 0.51–0.92), and 0.70 (95% CI: 0.52–0.94). These findings indicate a stable association between higher HALP levels and lower risk of incident comorbidity.

**Table 3:** Prospective associations between baseline HALP score and incident physical-cognitive comorbidity in the WCHAT cohort.

Model	HALP quartiles	HR (95% CI)	*P*-value
Model 1	Q1 (Ref)	1	-
	Q2	0.62 (0.46, 0.84)	0.002
	Q3	0.73 (0.54, 0.97)	0.031
	Q4	0.72 (0.54, 0.96)	0.026
Model 2	Q1 (Ref)	1	-
	Q2	0.60 (0.44, 0.81)	<0.001
	Q3	0.70 (0.52, 0.94)	0.017
	Q4	0.68 (0.51, 0.91)	0.010
Model 3	Q1 (Ref)	1	-
	Q2	0.60 (0.44, 0.81)	0.001
	Q3	0.69 (0.51, 0.92)	0.013
	Q4	0.70 (0.52, 0.94)	0.018

Among 2,782 participants free of physical-cognitive comorbidity at baseline, 330 incident events occurred during a median follow-up of 5 years (IQR: 3–5 years). Results are presented as hazard ratios (HRs) and 95% confidence intervals (CIs) from Cox proportional hazards models. Model 1 was unadjusted. Model 2 was adjusted for age and sex. Model 3 was fully adjusted for age, BMI, sex, education, race/ethnicity, smoking, alcohol use, hypertension, diabetes, coronary heart disease, and baseline single impairment status. HALP, hemoglobin-albumin-lymphocyte-platelet score.

**Figure 2:**
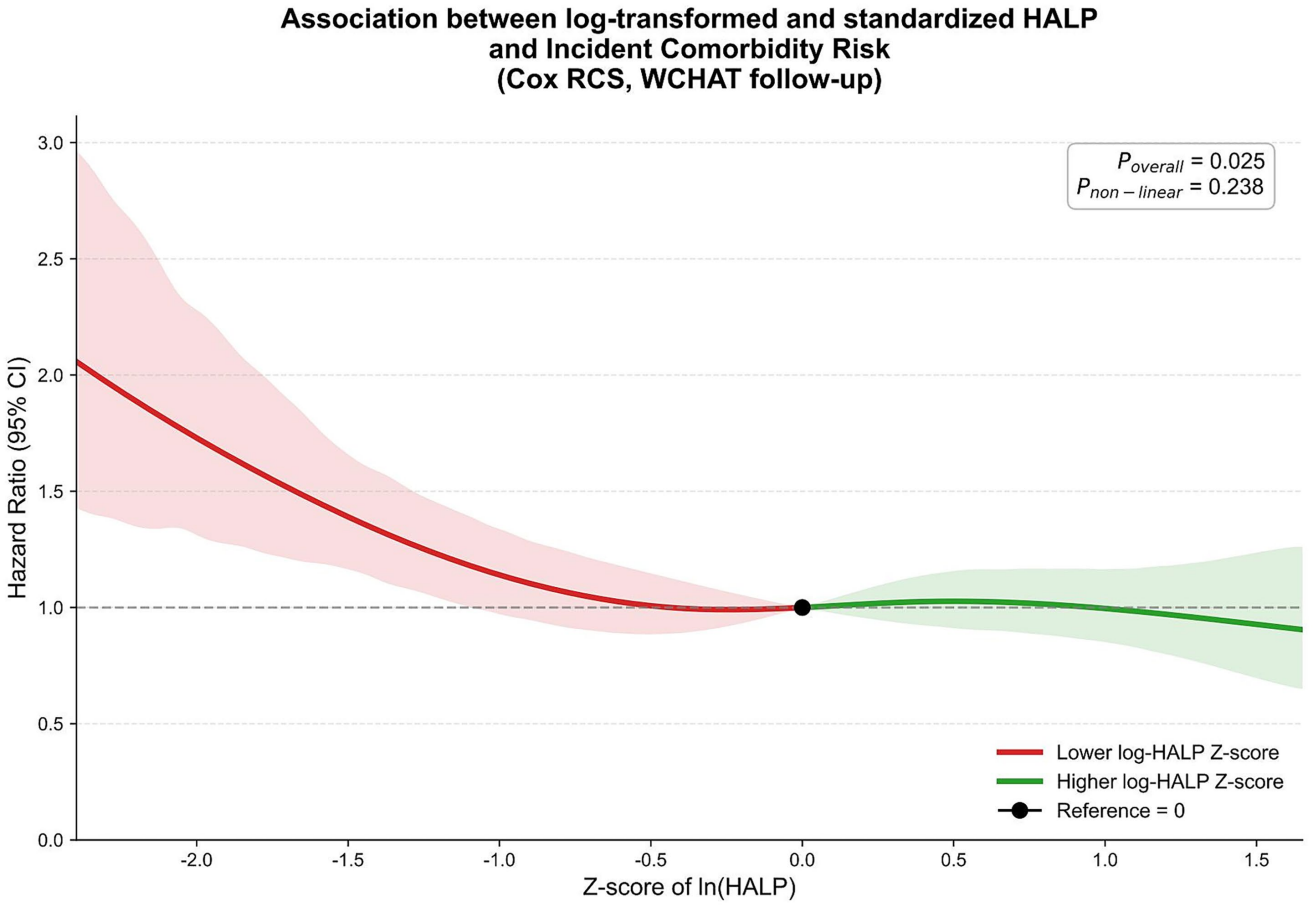
Restricted cubic spline association between log-transformed and standardized HALP and incident physical-cognitive comorbidity in the WCHAT cohort. Restricted cubic spline (RCS) analysis was performed using a Cox proportional hazards model to evaluate the association between baseline HALP and incident physical-cognitive comorbidity during follow-up in the WCHAT cohort. HALP was natural-log transformed and then standardized before RCS modeling. The x-axis is displayed as the *Z*-score of ln (HALP), consistent with the scale used for model fitting. The solid line represents the adjusted hazard ratio (HR), and the shaded area indicates the 95% confidence interval (CI). The reference value was set at 0 on the log-HALP *Z*-score scale, corresponding to HR = 1.00. Models were fully adjusted for age, body mass index, sex, education, race/ethnicity, smoking status, alcohol use, hypertension, diabetes, coronary heart disease, and baseline single impairment status. The overall association and nonlinearity were assessed by Wald tests.

When HALP was modeled as a continuous variable, the results remained robust (Supplementary Table S6). In the fully adjusted model, each 1-SD increase in HALP was associated with a 12% lower risk of incident physical-cognitive comorbidity (HR = 0.88, 95% CI: 0.79–0.99). RCS analysis using log-transformed and standardized HALP further supported these findings, showing a significant overall association with incident comorbidity risk (P for overall = 0.025), without evidence of significant nonlinearity (P for non-linearity = 0.238) (Figure 2). The RCS curve was displayed on the log-transformed and standardized HALP scale used for model fitting. In addition, no significant interactions were observed in the prospective subgroup analyses (Supplementary Table S5).

In sensitivity analyses excluding events that occurred within the first year of follow-up, the protective association for Q4 versus Q1 remained in WCHAT (HR = 0.60, 95% CI: 0.39–0.91) (Supplementary Table S7). In addition, in analyses restricted to participants aged ≥60 years, continuous HALP remained significantly associated with lower risk of incident physical-cognitive comorbidity in the fully adjusted model (HR = 0.85, 95% CI: 0.74–0.97) (Supplementary Table S8), supporting the robustness of the main results.

### Associations between HALP and all-cause mortality among participants with baseline comorbidity in UK biobank

**Figure 3:**
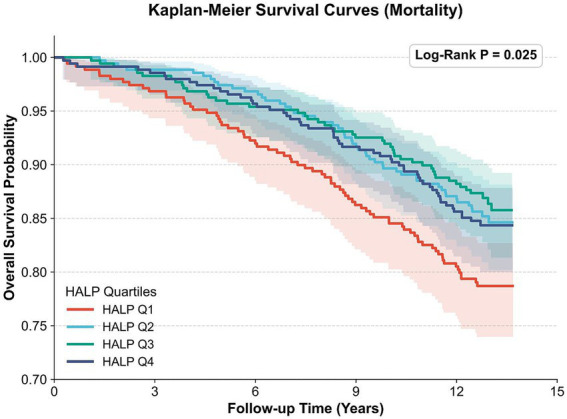
Kaplan–Meier survival curves for all-cause mortality among baseline physical-cognitive comorbidity participants stratified by HALP quartiles in the UK Biobank cohort. Participants were categorized into quartiles according to baseline HALP score. Overall survival differences across quartiles were assessed using the log-rank test. The overall log-rank *p* value is shown in the figure. Detailed post-hoc pairwise log-rank comparisons with Bonferroni correction are presented in Supplementary material.

In UK Biobank, 1,393 participants with baseline physical-cognitive comorbidity were included in the mortality analysis, during which 227 all-cause deaths occurred over a median follow-up of 14.48 years (IQR: 14.23–14.87 years). Kaplan–Meier survival curves are shown in Figure 3. Overall survival differed significantly across HALP quartiles (log-rank *p* = 0.025), with higher HALP groups showing a generally more favorable survival trend. Pairwise comparisons further showed that, after Bonferroni correction, only the comparison between Q1 and Q3 remained statistically significant (Supplementary Table S9).

Adjusted Cox regression results are shown in Table 4. After full adjustment, compared with Q1, Q3, and Q4 were associated with significantly lower all-cause mortality risks, with HRs of 0.68 (95% CI: 0.47–0.98) and 0.65 (95% CI: 0.45–0.93), respectively; Q2 showed the same direction of association but did not reach statistical significance. Continuous-variable analysis further supported these findings (Supplementary Table S10): in the fully adjusted model, each 1-SD increase in HALP was associated with a 20% lower risk of all-cause mortality (HR = 0.80, 95% CI: 0.70–0.92). Supplementary RCS analysis using log-transformed and standardized HALP showed a significant overall association with all-cause mortality (P for overall = 0.001), with no evidence of significant nonlinearity (P for non-linearity = 0.415) (Supplementary Figure S1). The RCS curve was displayed on the log-transformed and standardized HALP scale used for model fitting. No significant interactions were observed in the prospective subgroup analyses (Supplementary Table S5).

**Table 4:** Associations between baseline HALP score and all-cause mortality among participants with physical-cognitive comorbidity at baseline in the UK Biobank cohort.

Model	HALP quartiles	HR (95% CI)	*P*-value
Model 1	Q1 (Ref)	1	–
	Q2	0.67 (0.47, 0.95)	0.027
	Q3	0.60 (0.42, 0.87)	0.007
	Q4	0.70 (0.49, 1.00)	0.047
Model 2	Q1 (Ref)	1	–
	Q2	0.75 (0.53, 1.08)	0.120
	Q3	0.65 (0.45, 0.94)	0.023
	Q4	0.66 (0.46, 0.95)	0.024
Model 3	Q1 (Ref)	1	–
	Q2	0.82 (0.57, 1.18)	0.288
	Q3	0.68 (0.47, 0.98)	0.040
	Q4	0.65 (0.45, 0.93)	0.018

Among 1,393 UK Biobank participants with baseline physical-cognitive comorbidity, 227 deaths occurred during a median follow-up of 14.48 years (IQR: 14.23–14.87 years). Analysis was restricted to participants with physical-cognitive comorbidity at baseline. Results are presented as hazard ratios (HRs) and 95% confidence intervals (CIs) from Cox proportional hazards regression models. Model 1 was unadjusted. Model 2 was adjusted for age and sex. Model 3 was fully adjusted for age, BMI, sex, education, race/ethnicity, smoking, alcohol use, hypertension, diabetes, and coronary heart disease (CHD). HALP, hemoglobin-albumin-lymphocyte-platelet score.

In sensitivity analyses excluding deaths within the first year of follow-up, the protective association for Q4 versus Q1 remained in UK Biobank (HR = 0.63, 95% CI: 0.44–0.92) (Supplementary Table S7). In analyses restricted to participants aged ≥60 years, continuous HALP also remained significantly associated with lower all-cause mortality in the fully adjusted model (HR = 0.84, 95% CI: 0.72–0.98) (Supplementary Table S8), further supporting the robustness of the mortality findings.

### Exploratory and other supplementary analyses

To further address the asymmetry in the analytical framework, we conducted a supplementary exploratory incidence analysis among UK Biobank participants without baseline physical-cognitive comorbidity (Supplementary Table S11). In quartile analyses, only Q3 remained significantly associated with lower incident risk after full adjustment (HR = 0.48, 95% CI: 0.26–0.90), whereas continuous HALP did not reach statistical significance in the fully adjusted model (HR = 0.89, 95% CI: 0.72–1.11). These results should therefore be interpreted cautiously and are better regarded as supplementary evidence.

In addition, we evaluated the predictive performance of HALP-only Cox models using time-dependent ROC-AUC analysis. The highest time-dependent AUC was 0.5377 at 3 years for incident physical-cognitive comorbidity in WCHAT and 0.5835 at 10 years for all-cause mortality in UK Biobank (Supplementary Table S12). These values indicated limited standalone discrimination of HALP. After reanalysis using log-transformed and standardized HALP in the RCS models, no statistically significant nonlinearity or visually obvious turning point was observed; therefore, threshold effect analyses were not further performed.

## Discussion

Using two independent cohorts, WCHAT and UK Biobank, this study systematically evaluated the associations of the HALP score with the risk and prognosis of physical-cognitive comorbidity. The results showed that, in cross-sectional analyses, higher HALP scores were associated with lower risks of baseline physical-cognitive comorbidity; in WCHAT, higher baseline HALP scores were independently associated with lower risks of incident physical-cognitive comorbidity; and in UK Biobank participants with baseline physical-cognitive comorbidity, higher HALP scores were significantly associated with lower risks of all-cause mortality. These associations remained generally consistent in continuous-variable analyses, subgroup analyses, and sensitivity analyses, suggesting a relatively stable relationship between HALP and physical-cognitive comorbidity.

An important contribution of this study is the extension of HALP, a composite immune-nutritional index, to the complex geriatric phenotype of physical-cognitive comorbidity. Previous studies have mainly focused on the prognostic value of HALP in single disease settings, such as malignancies, cardiovascular disease, acute ischemic stroke, and post-stroke cognitive impairment, in which lower HALP scores often indicate poorer clinical outcomes or higher risks of adverse events (27–30). In contrast, the outcome examined in the present study is not a single organ-specific disease but a composite geriatric health outcome involving muscle strength, physical function, cognitive status, and long-term survival. Therefore, the significance of this study lies not only in expanding the application of HALP, but also in suggesting that HALP may reflect a more fundamental state of systemic biological vulnerability characterized by reduced immune-nutritional reserve, increased inflammatory burden, and impaired multisystem homeostasis.

From a mechanistic perspective, the individual components of HALP may jointly point to several key pathways involved in the development and progression of physical-cognitive comorbidity. First, lower hemoglobin levels indicate anemia and insufficient tissue oxygen supply. Previous studies have shown that anemia is associated with cognitive decline and elevated dementia risk, as well as poorer physical function, lower muscle strength, and worse physical performance (31–33). In middle-aged and older adults, chronic anemia may simultaneously affect cognition and physical function by reducing oxygen delivery efficiency, impairing cerebral energy metabolism, and aggravating fatigue and activity limitation. Second, the clinical significance of lower albumin levels extends beyond malnutrition alone. Low albumin typically reflects inadequate nutritional reserve and reduced anabolic capacity, and it is associated with lower muscle mass, poorer muscle strength, and reduced overall physiological reserve in middle-aged and older adults (34, 35). Prospective studies have shown that higher albumin levels are associated with lower risks of dementia, whereas low albumin combined with cognitive abnormality further increases the risk of all-cause mortality in middle-aged and older adults (36, 37). In addition, albumin is a negative acute-phase reactant that may decline further in inflammatory states, and thus may also reflect systemic frailty and increased inflammatory burden (38).

Furthermore, the lymphocyte and platelet components of HALP extend this index to the domains of inflammation and immune regulation. Reduced lymphocyte counts often indicate impaired immune function and immunosenescence, whereas platelets are involved not only in coagulation but also in inflammatory amplification, endothelial dysfunction, and thrombo-inflammatory responses (39, 40). Persistent chronic low-grade inflammation is considered an important shared pathway linking physical frailty and cognitive impairment and is associated with greater frailty severity, poorer clinical outcomes, and higher mortality risk (41, 42). Therefore, a low HALP score is more likely to reflect not an isolated laboratory abnormality, but a composite vulnerable state characterized by coexisting anemia, nutritional insufficiency, inflammatory activation, and immune imbalance. This may help explain why low HALP is associated not only with the development of physical-cognitive comorbidity but also with poorer survival after comorbidity has already formed.

From the perspective of disease course, this study captured the potential significance of HALP at both the pre-comorbidity and post-comorbidity stages. The prospective analysis in WCHAT suggests that HALP may provide meaningful risk information before physical-cognitive comorbidity becomes established, whereas the mortality analysis among UK Biobank participants with baseline comorbidity indicates that HALP remains closely related to long-term prognosis after comorbidity has developed. In other words, HALP may be associated not only with the occurrence of comorbidity but also with the disease burden and survival status after comorbidity formation. This finding complements previous studies that have primarily focused on adverse outcomes of cognitive frailty by suggesting that a composite index derived from routine laboratory tests may already carry meaningful risk information at an earlier stage. It should be noted, however, that this study did not symmetrically validate all outcomes in both cohorts. Rather, it leveraged the respective strengths of each cohort to focus on comorbidity formation in one and mortality prognosis after comorbidity in the other. Thus, the findings are better interpreted as complementary evidence regarding the direction of HALP-related associations across different stages of the disease spectrum rather than as results from two fully interchangeable validation samples.

From a clinical translation perspective, the main advantage of HALP is that it is derived entirely from routine hematologic and biochemical tests, making it easily obtainable, relatively low cost, and highly reproducible. These features support its practical feasibility for preliminary risk stratification in primary care and large population settings. In recent years, early prediction of adverse cognitive outcomes in middle-aged and older adults has received increasing attention. For example, a recent longitudinal study based on the CHARLS cohort used explainable machine-learning models to predict incident cognitive impairment, highlighting the growing importance of early identification of high-risk individuals in aging research (43). Compared with such multivariable, algorithm-driven prediction models, HALP is not a complex predictive system; rather, it is closer to a concise biological signal that can be embedded in routine clinical workflows. Nevertheless, time-dependent ROC-AUC analysis showed limited predictive performance of the HALP-only models, indicating that HALP should be interpreted as a complementary immune-nutritional marker rather than as a standalone predictive tool.

After natural-log transformation and standardization of HALP, the RCS analyses did not show evidence of statistically significant nonlinearity or visually obvious turning points for incident comorbidity in WCHAT or mortality in UK Biobank. Therefore, we did not attempt to define threshold values or clinical cutoffs for HALP. This supports interpreting HALP as a continuous immune-nutritional risk indicator rather than a marker with a specific decision threshold.

This study has several notable strengths. First, by using two independent cohorts, we examined HALP-related outcomes across three dimensions—cross-sectional prevalence, prospective incidence, and mortality prognosis—thereby providing a relatively comprehensive picture. Second, in the WCHAT incidence analysis, we additionally adjusted for baseline single impairment status, allowing a stricter distinction between progression from a single abnormality to a comorbid state. Third, we supplemented the main analyses with continuous-variable analyses, subgroup analyses, sensitivity analyses, comparisons between included and excluded participants, log-transformed and standardized RCS analyses, and predictive performance analyses, thereby more fully demonstrating the robustness and boundaries of the findings.

This study also has several limitations. First, as an observational study, residual confounding cannot be fully excluded despite adjustment for multiple potential confounders. In particular, potentially important factors such as cerebrovascular disease, which may affect both cognition and physical function, could not be incorporated into the unified main models because this variable was not systematically available in the baseline data of the self-established WCHAT cohort. Second, HALP was assessed only at baseline and therefore could not capture dynamic changes in immune-nutritional status over time. Third, in the WCHAT prospective analysis, included and excluded participants differed in multiple baseline characteristics, suggesting that sample selection and loss to follow-up may have introduced some degree of selection bias and that the findings should be generalized with caution. Fourth, the operational definitions of low grip strength and cognitive impairment were not fully identical between WCHAT and UK Biobank, and the two cohorts did not undertake fully symmetrical analytical tasks; accordingly, the findings are more appropriately interpreted in terms of direction of association, robustness, and biological consistency than by directly comparing absolute effect sizes. In addition, several statistically significant interactions were observed only in the cross-sectional subgroup analyses in UK Biobank, whereas no significant interactions were identified in the prospective subgroup analyses. These subgroup-specific differences may reflect variations in sample size, statistical power, and cohort characteristics, and therefore should be interpreted cautiously rather than overemphasized. Fifth, although we additionally conducted an exploratory incidence analysis in UK Biobank, the number of events in that analysis was relatively small and continuous HALP did not show a stable significant association, so these results should be regarded as supplementary evidence. Finally, the limited discrimination of the HALP-only models suggests that HALP may be better used as a component of integrated prediction models rather than as a stand-alone screening tool.

## Conclusion

Overall, this study suggests that higher HALP scores are associated with lower risks of physical-cognitive comorbidity and better survival prognosis. Although the HALP-only models showed limited discriminatory performance, HALP may still provide complementary information for immune-nutritional risk stratification by reflecting overall immune-nutritional reserve, inflammatory burden, and multisystem homeostasis in middle-aged and older adults. Further external validation in additional populations, together with repeated dynamic measurements, mechanistic studies, and integrated predictive modeling, will be needed to clarify the clinical utility of HALP as an auxiliary signal for risk stratification of physical-cognitive comorbidity.

## Data Availability

The datasets generated and analyzed during the current study are not publicly available at this time, as the West China Health and Aging Trend (WCHAT) database is newly established and currently supports ongoing longitudinal projects. Nevertheless, data from the WCHAT cohort are available from the corresponding author upon reasonable request. Data and materials for the UK Biobank cohort are available upon application to UK Biobank and can be accessed via the UK Biobank Research Analysis Platform (https://ukbiobank.dnanexus.com/panx/projects).

## Supplementary material

Supplementary Figure S1 Restricted cubic spline association between log-transformed and standardized HALP and all-cause mortality among participants with baseline physical-cognitive comorbidity in the UK Biobank cohort. Restricted cubic spline (RCS) analysis was performed using a Cox proportional hazards model to evaluate the association between baseline HALP and all-cause mortality among UK Biobank participants with baseline physical-cognitive comorbidity. HALP was natural-log transformed and then standardized before RCS modeling. The x-axis is displayed as the Z-score of ln(HALP), consistent with the scale used for model fitting. The solid line represents the adjusted hazard ratio (HR), and the shaded area indicates the 95% confidence interval (CI). The reference value was set at 0 on the log-HALP Z-score scale, corresponding to HR = 1.00. Models were fully adjusted for age, body mass index, sex, education, race/ethnicity, smoking status, alcohol use, hypertension, diabetes, and coronary heart disease. The overall association and nonlinearity were assessed by Wald tests.
