# Supplementary figures and images for: Association of the HALP score with baseline and incident physical-cognitive comorbidity and mortality: evidence from the WCHAT and UK Biobank cohorts
Source: Front Public Health. 2026 May 22;14:1842125. doi: 10.3389/fpubh.2026.1842125 (PMC13236514; doi:10.3389/fpubh.2026.1842125)

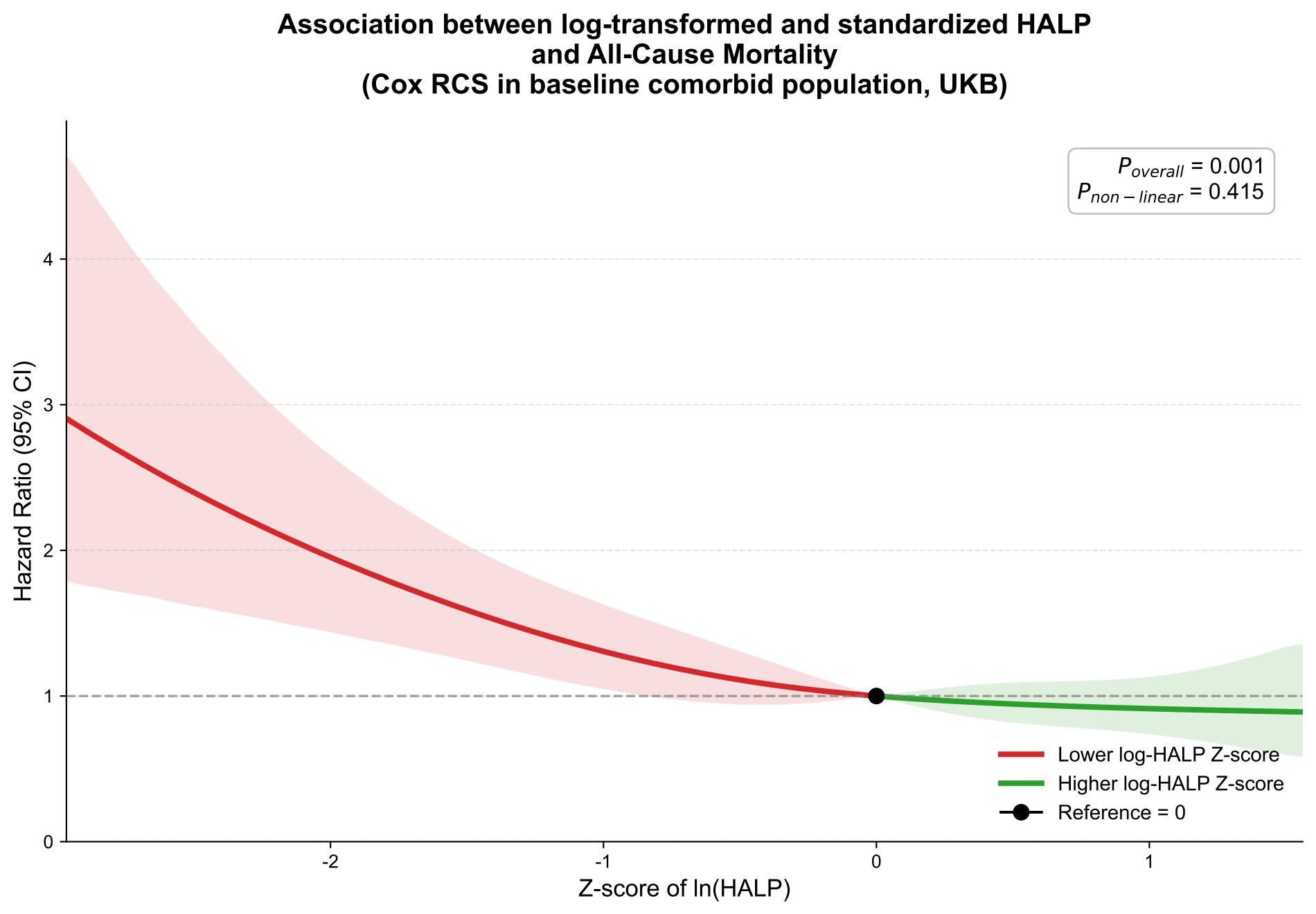

Supplement: Supplementary Figure S1 — Restricted cubic spline association between log-transformed and standardized HALP and all-cause mortality among participants with baseline physical-cognitive comorbidity in the UK Biobank cohort. Restricted cubic spline (RCS) analysis was performed using a Cox proportional hazards model to evaluate the association between baseline HALP and all-cause mortality among UK Biobank participants with baseline physical-cognitive comorbidity. HALP was natural-log transformed and then standardized before RCS modeling. The x-axis is displayed as the Z-score of ln(HALP), consistent with the scale used for model fitting. The solid line represents the adjusted hazard ratio (HR), and the shaded area indicates the 95% confidence interval (CI). The reference value was set at 0 on the log-HALP Z-score scale, corresponding to HR = 1.00. Models were fully adjusted for age, body mass index, sex, education, race/ethnicity, smoking status, alcohol use, hypertension, diabetes, and coronary heart disease. The overall association and nonlinearity were assessed by Wald tests. [file Image_1.jpg]
