# Supplementary material for: Association of the HALP score with baseline and incident physical-cognitive comorbidity and mortality: evidence from the WCHAT and UK Biobank cohorts
Source: Front Public Health. 2026 May 22;14:1842125. doi: 10.3389/fpubh.2026.1842125 (PMC13236514; doi:10.3389/fpubh.2026.1842125)
Supplement: Supplementary file 2 [file Data_Sheet_1.DOCX]

**Supplementary materials**

**Supplementary Methods**

**1. Exposure, covariates, and outcome definitions**

In the present study, the primary exposure was the HALP score, calculated from fasting blood samples using the following formula: HALP = (hemoglobin [g/L] × 10 × albumin [g/L] × lymphocytes [10^9/L]) / platelets [10^9/L]. Participants were categorized into quartiles (Q1–Q4) according to the cohort-specific distribution of the HALP score, with the lowest quartile (Q1) serving as the reference group. In supplementary analyses, HALP was also analyzed as a continuous variable and standardized as a Z score, with effect estimates reported per 1-standard deviation (SD) increase. For restricted cubic spline analyses, HALP was natural-log transformed and then standardized, as described below.

Physical-cognitive comorbidity was defined as the coexistence of low grip strength and cognitive impairment. In the WCHAT cohort, low grip strength was defined according to the Asian Working Group for Sarcopenia (AWGS) 2019 criteria (<28.0 kg for men and <18.0 kg for women), and cognitive impairment was defined as a Short Portable Mental Status Questionnaire (SPMSQ) score >2. In the UK Biobank (UKB) cohort, low grip strength was defined according to the European Working Group on Sarcopenia in Older People 2 (EWGSOP2) criteria (<27.0 kg for men and <16.0 kg for women), and cognitive impairment was operationally defined as a fluid intelligence score more than 1 SD below the mean of the baseline analytical sample. In both WCHAT and UK Biobank, maximum grip strength derived from both hands was used as the analytic grip strength variable.

Baseline covariates were collected using standardized procedures and included demographic characteristics (age, sex, educational level, and race/ethnicity), lifestyle factors (smoking and alcohol drinking status), body mass index (BMI), and self-reported chronic diseases (hypertension, diabetes, and coronary heart disease). In addition, for the WCHAT incidence analysis, baseline single impairment status, defined as the presence of either low grip strength alone or cognitive impairment alone, was additionally assessed and included in the fully adjusted model to account for pre-existing functional or cognitive vulnerability before the onset of physical-cognitive comorbidity.

**2. Logistic and Cox regression analyses**

We performed multivariable logistic regression models to estimate odds ratios (ORs) and 95% confidence intervals (CIs) for the cross-sectional associations between HALP and baseline physical-cognitive comorbidity in both the WCHAT and UK Biobank cohorts.

For the prospective analyses, Cox proportional hazards models were used to estimate hazard ratios (HRs) and 95% CIs for incident physical-cognitive comorbidity in the WCHAT cohort and for all-cause mortality among participants with baseline physical-cognitive comorbidity in the UK Biobank cohort. In addition, an exploratory incidence analysis was conducted among UK Biobank participants without baseline physical-cognitive comorbidity.

Three sequential models were fitted in the main analyses:

(1) Model 1, the crude model without adjustment;

(2) Model 2, adjusted for age and sex;

(3) Model 3, the fully adjusted model including age, BMI, sex, education, race/ethnicity, smoking, alcohol drinking, hypertension, diabetes, and coronary heart disease.

For the WCHAT prospective incidence analysis, Model 3 additionally included baseline single impairment status as an independent categorical covariate.

In the UK Biobank mortality analysis, Kaplan–Meier survival curves were generated to compare overall survival across HALP quartiles, and overall differences were assessed using the log-rank test. Pairwise post hoc log-rank comparisons between quartiles were further adjusted using the Bonferroni method.

**3. Restricted cubic spline analyses**

To explore potential nonlinear dose-response relationships between HALP and prospective outcomes, restricted cubic spline (RCS) models with four knots were fitted based on the fully adjusted Cox proportional hazards models. Considering the right-skewed distribution of HALP, HALP was first natural-log transformed to reduce skewness and the influence of extreme high values, and the log-transformed HALP was then standardized before RCS modeling. The RCS plots were presented on the same transformed scale used for model fitting, namely the Z-score of ln(HALP). For each RCS analysis, both the P value for the overall association and the P value for non-linearity were evaluated.

After reanalysis using log-transformed and standardized HALP, no statistically significant non-linearity or visually obvious turning point was observed. Therefore, exploratory threshold effect analyses were not further performed.

**4. Subgroup and sensitivity analyses**

Subgroup analyses were conducted to evaluate the consistency of associations across demographic and clinical strata. For cross-sectional analyses, subgroup-specific associations were estimated using logistic regression models. For prospective analyses, subgroup-specific associations were estimated using Cox proportional hazards models. Multiplicative interactions were tested by including an interaction term between HALP and the corresponding stratification variable. Stratification variables included age, sex, BMI, smoking, alcohol drinking, and chronic disease status.

Sensitivity analyses were conducted by excluding outcome events occurring within the first year of follow-up to reduce potential reverse causation. Additional restricted analyses were also performed among participants aged ≥60 years to evaluate the robustness of the primary age threshold used in the main analyses.

**5. Comparison between included and excluded participants**

To assess potential selection bias, we compared baseline characteristics between participants included in and excluded from the prospective analyses. In WCHAT, this comparison was performed for the incidence analysis cohort after excluding participants with baseline physical-cognitive comorbidity. In UK Biobank, a similar comparison was performed for the exploratory incidence analysis cohort.

**6. Predictive performance analysis**

To evaluate the predictive performance boundary of HALP as a single indicator, HALP-only Cox proportional hazards models were fitted for the WCHAT incidence analysis and the UK Biobank mortality analysis. Model discrimination was evaluated using Harrell’s C-index and time-dependent receiver operating characteristic curve analysis. Time-dependent area under the curve (AUC) values were calculated at prespecified time points. These analyses were intended to clarify whether HALP should be interpreted as a standalone predictive tool or rather as a complementary marker for immune-nutritional risk assessment.

**Table S1. Missingness of HALP components, derived HALP, covariates, and participant-level exclusion summaries in the WCHAT and UK Biobank cohorts**

**Panel A. Missingness of variables**

| Variable | WCHAT Cohort |  | UK Biobank Cohort |  |
| --- | --- | --- | --- | --- |
|  | Raw source population (N = 7,536)n  (%) missing | Age ≥ 50 population (N = 7,439)n  (%) missing | Raw source population (N = 502,357)n  (%) missing | Age ≥ 50 population (N = 384,547)n  (%) missing |
| Hemoglobin | 420 (5.57%) | 412 (5.54%) | 24,323 (4.84%) | 18,254 (4.75%) |
| Albumin | 433 (5.75%) | 425 (5.71%) | 72,404 (14.41%) | 55,096 (14.33%) |
| Lymphocytes | 416 (5.52%) | 408 (5.48%) | 25,212 (5.02%) | 18,958 (4.93%) |
| Platelets | 423 (5.61%) | 415 (5.58%) | 24,326 (4.84%) | 18,256 (4.75%) |
| HALP | 452 (6.00%) | 446 (6.00%) | 84,186 (16.76%) | 61,109 (15.89%) |
| Age | 0 (0.00%) | 0 (0.00%) | 0 (0.00%) | 0 (0.00%) |
| Sex | 0 (0.00%) | 0 (0.00%) | 0 (0.00%) | 0 (0.00%) |
| Race / ethnicity | 0 (0.00%) | 0 (0.00%) | 899 (0.18%) | 640 (0.17%) |
| BMI | 144 (1.91%) | 142 (1.91%) | 3,105 (0.62%) | 2,342 (0.61%) |
| Education | 90 (1.19%) | 89 (1.20%) | 0 (0.00%) | 0 (0.00%) |
| Smoking | 118 (1.57%) | 115 (1.55%) | 2,949 (0.59%) | 2,327 (0.61%) |
| Alcohol use | 96 (1.27%) | 94 (1.26%) | 1,653 (0.33%) | 1,168 (0.30%) |
| Hypertension | 47 (0.62%) | 47 (0.63%) | 0 (0.00%) | 0 (0.00%) |
| Diabetes | 53 (0.70%) | 52 (0.70%) | 2,616 (0.52%) | 1,853 (0.48%) |
| Coronary heart disease | 62 (0.82%) | 61 (0.82%) | 0 (0.00%) | 0 (0.00%) |
| Cognitive assessment* | 219 (2.91%) | 215 (2.89%) | 336,949 (67.07%) | 257,231 (66.89%) |
| Left grip strength | 450 (5.97%) | 442 (5.94%) | 3,411 (0.68%) | 2,636 (0.69%) |
| Right grip strength | 446 (5.92%) | 438 (5.89%) | 3,344 (0.67%) | 2,550 (0.66%) |
| Maximum grip strength | 420 (5.57%) | 412 (5.54%) | 2,362 (0.47%) | 1,748 (0.45%) |

**Panel B. Definition of extreme HALP values**

| Metric | WCHAT Cohort | UK Biobank Cohort |
| --- | --- | --- |
| 1st percentile cutoff | 22.9 | 17.95 |
| Number of HALP values below the 1st percentile | 76 | 3,206 |
| 99th percentile cutoff | 195.29 | 126.29 |
| Number of HALP values above the 99th percentile | 76 | 3,206 |

**Panel C. Participant-level exclusion summary**

| Metric | WCHAT Cohort | UK Biobank Cohort |
| --- | --- | --- |
| Participants excluded because of missing or extreme HALP | 598 | 67,521 |
| Participants excluded because of any missing baseline covariate | 356 | 965 |
| Participants excluded because of missing baseline functional tests / date fields^†^ | 528 | 214,406 |

Note: Missingness proportions are summarized in both the raw source population and the age ≥ 50 population used for subsequent screening. Extreme HALP values were defined as values below the 1st percentile or above the 99th percentile of the cohort-specific HALP distribution. Grip-strength completeness for participant-level exclusion was based on maximum grip strength rather than either single-hand measurement alone. Participants with only one available hand measurement were retained if maximum grip strength could still be calculated/derived. HALP, hemoglobin-albumin-lymphocyte-platelet score; BMI, body mass index.

·Cognitive assessment refers to the baseline cognitive score in the WCHAT cohort and the fluid intelligence score in the UK Biobank cohort.

† In the WCHAT cohort, exclusion because of missing functional tests was determined from the baseline cognitive score and maximum grip strength. In the UK Biobank cohort, exclusion was based on the derived maximum grip strength, fluid intelligence score, and baseline assessment date.

**Table S2. Comparison between included and excluded participants in the WCHAT incidence analysis and the exploratory UK Biobank incidence analysis**

| Variable | WCHAT Cohort |  |  | UK Biobank Cohort |  |  |
| --- | --- | --- | --- | --- | --- | --- |
|  | Included (n = 2,782) | Excluded (n = 2,355) | P-value | Included (n = 14,050) | Excluded (n = 86,212) | P-value |
| Age (years) | 61.93 ± 7.76 | 61.25 ± 8.14 | 0.002 | 58.92 ± 5.15 | 60.43 ± 5.41 | <0.001 |
| BMI (kg/m²) | 25.45 ± 3.75 | 25.73 ± 4.28 | 0.013 | 26.84 ± 4.26 | 27.57 ± 4.77 | <0.001 |
| HALP score | 75.58 ± 30.36 | 71.04 ± 31.40 | <0.001 | 54.49 ± 18.95 | 54.70 ± 19.64 | 0.221 |
| Cognitive score | 0.88 ± 1.24 | 0.91 ± 1.37 | 0.361 | 6.75 ± 2.02 | 5.93 ± 2.10 | <0.001 |
| Grip strength (kg) | 23.60 ± 8.43 | 23.46 ± 9.06 | 0.554 | 33.30 ± 10.89 | 30.79 ± 10.63 | <0.001 |
| Sex, n (%) |  |  | 0.011 |  |  | <0.001 |
| Female | 1,736 (62.4%) | 1,387 (58.9%) |  | 6,880 (49.0%) | 47,088 (54.6%) |  |
| Male | 1,046 (37.6%) | 968 (41.1%) |  | 7,170 (51.0%) | 39,124 (45.4%) |  |
| Education, n (%) |  |  | <0.001 |  |  | <0.001 |
| Below junior high school | 1,680 (60.4%) | 1,211 (51.4%) |  | 2,620 (18.6%) | 27,955 (32.4%) |  |
| Junior high school and above | 1,102 (39.6%) | 1,144 (48.6%) |  | 11,430 (81.4%) | 58,257 (67.6%) |  |
| Race, n (%) |  |  | <0.001 |  |  | <0.001 |
| Recognized minority group | 1,349 (48.5%) | 739 (31.4%) |  | 1,149 (8.2%) | 10,494 (12.2%) |  |
| Majority / dominant group | 1,433 (51.5%) | 1,616 (68.6%) |  | 12,901 (91.8%) | 75,718 (87.8%) |  |
| Smoking, n (%) |  |  | <0.001 |  |  | <0.001 |
| No | 2,282 (82.0%) | 1,844 (78.3%) |  | 13,368 (95.1%) | 78,307 (90.8%) |  |
| Yes | 500 (18.0%) | 511 (21.7%) |  | 682 (4.9%) | 7,905 (9.2%) |  |
| Alcohol drinking, n (%) |  |  | <0.001 |  |  | <0.001 |
| No | 2,163 (77.7%) | 1,949 (82.8%) |  | 621 (4.4%) | 7,073 (8.2%) |  |
| Yes | 619 (22.3%) | 406 (17.2%) |  | 13,429 (95.6%) | 79,139 (91.8%) |  |
| Hypertension, n (%) |  |  | <0.001 |  |  | <0.001 |
| No | 1,190 (42.8%) | 1,127 (47.9%) |  | 10,641 (75.7%) | 58,889 (68.3%) |  |
| Yes | 1,592 (57.2%) | 1,228 (52.1%) |  | 3,409 (24.3%) | 27,323 (31.7%) |  |
| Diabetes, n (%) |  |  | 0.701 |  |  | <0.001 |
| No | 2,383 (85.7%) | 2,027 (86.1%) |  | 13,556 (96.5%) | 80,732 (93.6%) |  |
| Yes | 399 (14.3%) | 328 (13.9%) |  | 494 (3.5%) | 5,480 (6.4%) |  |
| Coronary heart disease, n (%) |  |  | 0.003 |  |  | <0.001 |
| No | 2,595 (93.3%) | 2,144 (91.0%) |  | 13,607 (96.8%) | 81,586 (94.6%) |  |
| Yes | 187 (6.7%) | 211 (9.0%) |  | 443 (3.2%) | 4,626 (5.4%) |  |

Note: The WCHAT cohort columns compare participants included in the prospective incidence analysis with those excluded after baseline non-comorbidity screening because of loss to follow-up or unavailable outcome assessment. The UK Biobank cohort columns compare participants included in the exploratory incidence analysis with those excluded because of unavailable repeated grip strength, repeated cognitive assessment, or usable follow-up time. Continuous variables are presented as mean ± standard deviation, and categorical variables are presented as number (percentage). Cognitive score indicates the Short Portable Mental Status Questionnaire (SPMSQ) score in the WCHAT cohort and the Fluid Intelligence score in the UK Biobank cohort. Grip strength indicates maximum grip strength derived from both hands in both the WCHAT cohort and the UK Biobank cohort. BMI, body mass index; HALP, hemoglobin-albumin-lymphocyte-platelet score.

**Table S3. Descriptive comparison of physical-cognitive comorbidity prevalence and follow-up event rates under cohort-specific operational definitions in the WCHAT and UK Biobank cohorts**

| Cohort | Analysis Stage | Low Grip Strength Definition | Cognitive Impairment Definition | Analytical Sample Size (n) | Cases / Events  (n) | Rate  (%) |
| --- | --- | --- | --- | --- | --- | --- |
| WCHAT | Baseline cross-sectional comorbidity | AWGS 2019:Male < 28.0 kgFemale < 18.0 kg | SPMSQ > 2 | 5,957 | 820 | 13.8 |
|  | Prospective incidence of physical-cognitive comorbidity | AWGS 2019:Male < 28.0 kgFemale < 18.0 kg | SPMSQ > 2 | 2,782 | 330 | 11.9 |
| UK Biobank | Baseline cross-sectional comorbidity | EWGSOP2 (maximum grip strength):Male < 27.0 kgFemale < 16.0 kg | Fluid intelligence score < 1 SD below the mean of the baseline analytical sample | 101,655 | 1,393 | 1.4 |
|  | Exploratory incidence of physical-cognitive comorbidity | EWGSOP2 (maximum grip strength):Male < 27.0 kgFemale < 16.0 kg | Fluid intelligence score < 1 SD below the mean of the baseline analytical sample | 14,050 | 93 | 0.7 |
|  | All-cause mortality among baseline comorbid participants | Defined as above | Defined as above | 1,393 | 227 | 16.3 |

Note: This table summarizes the baseline prevalence and follow-up event rates of physical-cognitive comorbidity under the cohort-specific operational definitions used in the present study. In the WCHAT cohort, low grip strength was defined according to AWGS 2019, and cognitive impairment was defined as SPMSQ > 2. In the UK Biobank cohort, low grip strength was defined according to EWGSOP2 using maximum grip strength, and cognitive impairment was operationally defined as a fluid intelligence score below 1 standard deviation of the baseline analytical sample mean. Rates are provided for descriptive comparison only. Because the operational definitions differed between cohorts, absolute prevalence, incidence, and prognosis estimates should not be interpreted as directly comparable across cohorts. AWGS, Asian Working Group for Sarcopenia; EWGSOP2, European Working Group on Sarcopenia in Older People 2; SPMSQ, Short Portable Mental Status Questionnaire; SD, standard deviation.

**Table S4. Cross-sectional associations of continuous HALP with baseline physical-cognitive comorbidity in the WCHAT and UK Biobank cohorts**

| Model | Variable | WCHAT Cohort OR (95% CI) | P-value | UK Biobank Cohort OR (95% CI) | P-value |
| --- | --- | --- | --- | --- | --- |
| Model 1 | HALP per 1-SD increase | 0.77 (0.71, 0.84) | <0.001 | 0.99 (0.93, 1.04) | 0.595 |
| Model 2 | HALP per 1-SD increase | 0.82 (0.75, 0.89) | <0.001 | 1.00 (0.95, 1.06) | 0.996 |
| Model 3 | HALP per 1-SD increase | 0.91 (0.84, 0.99) | 0.034 | 0.92 (0.87, 0.97) | 0.002 |

Note: Results are presented as odds ratios (ORs) and 95% confidence intervals (CIs). Model 1 was unadjusted. Model 2 was adjusted for age and sex. Model 3 was fully adjusted for age, BMI, sex, education, race/ethnicity, smoking, alcohol use, hypertension, diabetes, and coronary heart disease (CHD). Continuous HALP was analyzed per 1-standard deviation increase.

**Table S5. Subgroup analyses of the associations between continuous HALP and study outcomes in the WCHAT and UK Biobank cohorts**

**Panel A. Cross-sectional associations with baseline physical-cognitive comorbidity**

| Subgroup | Stratum | WCHAT N | WCHAT Events | WCHAT OR (95% CI) | P for int. | UK Biobank N | UKB Events | UKB OR (95% CI) | P for int. |
| --- | --- | --- | --- | --- | --- | --- | --- | --- | --- |
| Age | <65 | 3,683 | 337 | 0.91 (0.80, 1.04) | 0.681 | 75,517 | 866 | 0.94 (0.88, 1.01) | 0.102 |
|  | ≥65 | 2,274 | 483 | 0.92 (0.82, 1.02) |  | 26,138 | 527 | 0.88 (0.81, 0.97) |  |
| Sex | Female | 3,745 | 622 | 0.92 (0.83, 1.02) | 0.536 | 54,771 | 803 | 0.90 (0.84, 0.97) | 0.532 |
|  | Male | 2,212 | 198 | 0.89 (0.76, 1.05) |  | 46,884 | 590 | 0.94 (0.87, 1.02) |  |
| Education | Low | 3,654 | 763 | 0.91 (0.83, 1.00) | 0.994 | 31,464 | 889 | 0.89 (0.83, 0.95) | 0.019 |
|  | High | 2,303 | 57 | 0.93 (0.70, 1.23) |  | 70,191 | 504 | 0.97 (0.88, 1.06) |  |
| BMI | <24 / <25 | 2,302 | 415 | 0.87 (0.76, 0.98) | 0.26 | 32,696 | 382 | 1.01 (0.91, 1.12) | 0.003 |
|  | ≥24 / ≥25 | 3,655 | 405 | 0.96 (0.86, 1.08) |  | 68,959 | 1,011 | 0.90 (0.84, 0.96) |  |
| Smoking | No | 4,838 | 712 | 0.91 (0.83, 1.00) | 0.701 | 92,892 | 1,217 | 0.90 (0.85, 0.95) | 0.117 |
|  | Yes | 1,119 | 108 | 0.92 (0.73, 1.15) |  | 8,763 | 176 | 1.03 (0.90, 1.19) |  |
| Alcohol | No | 4,830 | 718 | 0.89 (0.82, 0.98) | 0.408 | 8,017 | 323 | 0.96 (0.86, 1.08) | 0.385 |
|  | Yes | 1,127 | 102 | 1.09 (0.87, 1.37) |  | 93,638 | 1,070 | 0.90 (0.85, 0.96) |  |
| Hypertension | No | 2,696 | 379 | 0.96 (0.84, 1.09) | 0.666 | 70,344 | 814 | 0.96 (0.89, 1.03) | 0.015 |
|  | Yes | 3,261 | 441 | 0.88 (0.79, 0.99) |  | 31,311 | 579 | 0.86 (0.79, 0.93) |  |
| Diabetes | No | 5,140 | 730 | 0.91 (0.83, 1.00) | 0.9 | 95,460 | 1,172 | 0.93 (0.87, 0.98) | 0.531 |
|  | Yes | 817 | 90 | 0.92 (0.72, 1.18) |  | 6,195 | 221 | 0.86 (0.76, 0.98) |  |
| CHD | No | 5,482 | 743 | 0.91 (0.83, 1.00) | 0.55 | 96,416 | 1,223 | 0.93 (0.88, 0.98) | 0.251 |
|  | Yes | 475 | 77 | 0.97 (0.73, 1.30) |  | 5,239 | 170 | 0.85 (0.73, 0.99) |  |

**Panel B. Prospective associations with follow-up outcomes**

| Subgroup | Stratum | WCHAT N | WCHAT Events | WCHAT HR (95% CI) | P for int. | UK Biobank N | UKB Events | UKB HR (95% CI) | P for int. |
| --- | --- | --- | --- | --- | --- | --- | --- | --- | --- |
| Age | <65 | 1,779 | 128 | 0.95 (0.79–1.14) | 0.348 | 866 | 107 | 0.83 (0.68–1.02) | 0.598 |
|  | ≥65 | 1,003 | 202 | 0.85 (0.73–0.99) |  | 527 | 120 | 0.78 (0.64–0.94) |  |
| Sex | Female | 1,736 | 209 | 0.88 (0.76–1.02) | 0.89 | 803 | 104 | 0.94 (0.75–1.18) | 0.124 |
|  | Male | 1,046 | 121 | 0.86 (0.71–1.05) |  | 590 | 123 | 0.73 (0.62–0.87) |  |
| BMI | <24 / <25 | 1,016 | 139 | 0.93 (0.78–1.11) | 0.67 | 382 | 58 | 0.80 (0.60–1.07) | 0.663 |
|  | ≥24 / ≥25 | 1,766 | 191 | 0.88 (0.75–1.02) |  | 1,011 | 169 | 0.83 (0.71–0.98) |  |
| Smoking | No | 2,282 | 265 | 0.89 (0.78–1.02) | 0.686 | 1,217 | 183 | 0.81 (0.69–0.95) | 0.788 |
|  | Yes | 500 | 65 | 0.82 (0.62–1.10) |  | 176 | 44 | 0.78 (0.59–1.03) |  |
| Alcohol | No | 2,163 | 262 | 0.84 (0.73–0.96) | 0.096 | 323 | 55 | 0.67 (0.51–0.88) | 0.134 |
|  | Yes | 619 | 68 | 1.06 (0.83–1.36) |  | 1,070 | 172 | 0.85 (0.73–1.00) |  |
| Hypertension | No | 1,190 | 127 | 0.85 (0.71–1.04) | 0.586 | 814 | 107 | 0.86 (0.71–1.05) | 0.303 |
|  | Yes | 1,592 | 203 | 0.91 (0.78–1.05) |  | 579 | 120 | 0.74 (0.61–0.90) |  |
| Diabetes | No | 2,383 | 288 | 0.91 (0.80–1.03) | 0.172 | 1,172 | 169 | 0.80 (0.68–0.94) | 0.986 |
|  | Yes | 399 | 42 | 0.75 (0.54–1.04) |  | 221 | 58 | 0.83 (0.63–1.09) |  |
| CHD | No | 2,595 | 302 | 0.89 (0.79–1.01) | 0.426 | 1,223 | 188 | 0.83 (0.71–0.96) | 0.436 |
|  | Yes | 187 | 28 | 0.73 (0.47–1.14) |  | 170 | 39 | 0.75 (0.54–1.05) |  |

Note: Results are presented as odds ratios (ORs) for cross-sectional analyses and hazard ratios (HRs) for prospective analyses, with corresponding 95% confidence intervals (CIs), analyzed per 1-standard deviation increase in the continuous HALP score. In Panel A, the outcome was baseline physical-cognitive comorbidity in both cohorts. In Panel B, the prospective outcome for the WCHAT cohort was incident physical-cognitive comorbidity, while the outcome for the UK Biobank cohort was all-cause mortality among participants with physical-cognitive comorbidity at baseline. BMI cutoffs were <24 and ≥24 kg/m² for WCHAT and <25 and ≥25 kg/m² for UK Biobank. P for int. indicates the P-value for multiplicative interaction between the HALP score and the subgroup variable. HALP, hemoglobin-albumin-lymphocyte-platelet score; BMI, body mass index; CHD, coronary heart disease.

**Table S6. Prospective associations of continuous HALP with incident physical-cognitive comorbidity in the WCHAT cohort**

| Model | Variable | HR (95% CI) | P-value |
| --- | --- | --- | --- |
| Model 1 | HALP per 1-SD increase | 0.89 (0.79, 1.00) | 0.043 |
| Model 2 | HALP per 1-SD increase | 0.88 (0.78, 0.98) | 0.023 |
| Model 3 | HALP per 1-SD increase | 0.88 (0.79, 0.99) | 0.040 |

Note: Results are presented as hazard ratios (HRs) and 95% confidence intervals (CIs) from Cox proportional hazards regression models. Model 1 was unadjusted. Model 2 was adjusted for age and sex. Model 3 was fully adjusted according to the covariate specification used in the final WCHAT incidence model. Continuous HALP was analyzed per 1-standard deviation increase. HALP, hemoglobin-albumin-lymphocyte-platelet score. The WCHAT incidence cohort included 2,782 participants with 330 incident events.

**Table S7. Sensitivity analyses of the associations between baseline HALP score and study outcomes after excluding events occurring within the first year of follow-up**

| Model | HALP Quartiles | WCHAT Cohort (Incident Comorbidity) |  | UK Biobank Cohort (All-cause Mortality) |  |
| --- | --- | --- | --- | --- | --- |
|  |  | HR (95% CI) | P-value | HR (95% CI) | P-value |
| Model 1 | Q1 (Ref) | 1 | – | 1 | – |
|  | Q2 | 0.67 (0.45, 1.00) | 0.05 | 0.69 (0.48, 0.99) | 0.042 |
|  | Q3 | 0.90 (0.62, 1.31) | 0.588 | 0.63 (0.44, 0.92) | 0.016 |
|  | Q4 | 0.66 (0.44, 0.99) | 0.046 | 0.69 (0.48, 0.99) | 0.042 |
| Model 2 | Q1 (Ref) | 1 | – | 1 | – |
|  | Q2 | 0.63 (0.42, 0.95) | 0.026 | 0.78 (0.54, 1.12) | 0.177 |
|  | Q3 | 0.83 (0.57, 1.21) | 0.337 | 0.69 (0.48, 1.00) | 0.048 |
|  | Q4 | 0.61 (0.40, 0.92) | 0.019 | 0.66 (0.46, 0.95) | 0.024 |
| Model 3 | Q1 (Ref) | 1 | – | 1 | – |
|  | Q2 | 0.62 (0.42, 0.94) | 0.023 | 0.84 (0.58, 1.21) | 0.355 |
|  | Q3 | 0.79 (0.54, 1.15) | 0.217 | 0.71 (0.49, 1.03) | 0.073 |
|  | Q4 | 0.60 (0.39, 0.91) | 0.016 | 0.63 (0.44, 0.92) | 0.016 |

Note: Analysis was restricted to events occurring after the first year of follow-up to minimize potential reverse causation. Results are presented as hazard ratios (HRs) and 95% confidence intervals (CIs) from Cox proportional hazards regression models. Model 1 was unadjusted. Model 2 was adjusted for age and sex. For the WCHAT cohort, Model 3 was fully adjusted for age, body mass index, sex, education, race/ethnicity, smoking, alcohol drinking, hypertension, diabetes, coronary heart disease, and baseline single impairment status. For the UK Biobank cohort, Model 3 was fully adjusted for age, body mass index, sex, education, race/ethnicity, smoking, alcohol drinking, hypertension, diabetes, and coronary heart disease. HALP, hemoglobin-albumin-lymphocyte-platelet score.

**Table S8. Age-restricted sensitivity analyses of continuous HALP in the WCHAT and UK Biobank cohorts**

| Model | Variable | WCHAT Cohort  (Incident Comorbidity, aged ≥ 60) |  | UK Biobank Cohort  (All-cause Mortality, aged ≥ 60) |  |
| --- | --- | --- | --- | --- | --- |
|  |  | HR (95% CI) | P-value | HR (95% CI) | P-value |
| Model 1 | HALP per 1-SD increase | 0.82 (0.72, 0.94) | 0.003 | 0.89 (0.76, 1.04) | 0.13 |
| Model 2 | HALP per 1-SD increase | 0.84 (0.74, 0.96) | 0.009 | 0.85 (0.73, 1.00) | 0.043 |
| Model 3 | HALP per 1-SD increase | 0.85 (0.74, 0.97) | 0.015 | 0.84 (0.72, 0.98) | 0.029 |

Note: Results are presented as hazard ratios (HRs) and 95% confidence intervals (CIs) from Cox proportional hazards regression models analyzed per 1-standard deviation increase in the continuous HALP score. The analysis was restricted to participants aged ≥ 60 years. For the WCHAT cohort, the outcome was incident physical-cognitive comorbidity; for the UK Biobank cohort, the outcome was all-cause mortality among baseline physical-cognitive comorbidity participants. Model 1 was unadjusted. Model 2 was adjusted for age and sex. Model 3 was fully adjusted for age, BMI, sex, education, race/ethnicity, smoking, alcohol use, hypertension, diabetes, and coronary heart disease; for the WCHAT cohort, baseline single impairment status was additionally included in the fully adjusted model.HALP, hemoglobin-albumin-lymphocyte-platelet score.

**Table S9. Post-hoc pairwise log-rank comparisons for all-cause mortality across HALP quartiles in the UK Biobank cohort**

| Comparison | Log-rank χ² | P-value | Bonferroni-corrected P-value |
| --- | --- | --- | --- |
| Q1 vs Q2 | 4.9844 | 0.026 | 0.153 |
| Q1 vs Q3 | 7.4382 | 0.006 | 0.038 |
| Q1 vs Q4 | 4.0312 | 0.045 | 0.268 |
| Q2 vs Q3 | 0.2758 | 0.599 | ＞0.999 |
| Q2 vs Q4 | 0.0498 | 0.823 | ＞0.999 |
| Q3 vs Q4 | 0.5509 | 0.458 | ＞0.999 |

Note: Pairwise post-hoc comparisons were performed using log-rank tests between HALP quartile groups in the UK Biobank baseline comorbid population. Bonferroni correction was applied for 6 pairwise comparisons. The overall Kaplan–Meier comparison across quartiles showed a significant difference in survival probability (log-rank P = 0.025). After Bonferroni correction, only the comparison between Q1 and Q3 remained statistically significant. HALP, hemoglobin-albumin-lymphocyte-platelet score.

**Table S10. Associations of continuous HALP with all-cause mortality among baseline physical-cognitive comorbidity participants in the UK Biobank cohort**

| Model | Variable | HR (95% CI) | P-value |
| --- | --- | --- | --- |
| Model 1 | HALP per 1-SD increase | 0.84 (0.73, 0.96) | 0.012 |
| Model 2 | HALP per 1-SD increase | 0.82 (0.71, 0.94) | 0.004 |
| Model 3 | HALP per 1-SD increase | 0.80 (0.70, 0.92) | 0.002 |

Note: Results are presented as hazard ratios (HRs) and 95% confidence intervals (CIs) from Cox proportional hazards regression models. Model 1 was unadjusted. Model 2 was adjusted for age and sex. Model 3 was fully adjusted for age, BMI, sex, education, race/ethnicity, smoking, alcohol use, hypertension, diabetes, and coronary heart disease (CHD). Continuous HALP was analyzed per 1-standard deviation increase. HALP, hemoglobin-albumin-lymphocyte-platelet score.

**Table S11. Exploratory associations between baseline HALP score and incident physical-cognitive comorbidity in the UK Biobank cohort**

**Panel A. HALP quartiles**

| Model | HALP Quartiles | HR (95% CI) | P-value |
| --- | --- | --- | --- |
| Model 1 | Q1 (Ref) | 1 | – |
|  | Q2 | 0.76 (0.44, 1.31) | 0.324 |
|  | Q3 | 0.53 (0.29, 0.99) | 0.047 |
|  | Q4 | 0.93 (0.55, 1.57) | 0.781 |
| Model 2 | Q1 (Ref) | 1 | – |
|  | Q2 | 0.75 (0.43, 1.30) | 0.301 |
|  | Q3 | 0.52 (0.28, 0.97) | 0.04 |
|  | Q4 | 0.92 (0.54, 1.56) | 0.754 |
| Model 3 | Q1 (Ref) | 1 | – |
|  | Q2 | 0.74 (0.43, 1.29) | 0.286 |
|  | Q3 | 0.48 (0.26, 0.90) | 0.023 |
|  | Q4 | 0.78 (0.45, 1.34) | 0.364 |

**Panel B. Continuous HALP**

| Model | Variable | HR (95% CI) | P-value |
| --- | --- | --- | --- |
| Model 1 | HALP per 1-SD increase | 0.97 (0.79, 1.20) | 0.807 |
| Model 2 | HALP per 1-SD increase | 0.98 (0.79, 1.20) | 0.821 |
| Model 3 | HALP per 1-SD increase | 0.89 (0.72, 1.11) | 0.309 |

Note: Results are presented as hazard ratios (HRs) and 95% confidence intervals (CIs) from Cox proportional hazards regression models. This analysis was exploratory and based on participants without baseline physical-cognitive comorbidity in the UK Biobank cohort who had available repeated grip strength, repeated cognitive assessment, and usable follow-up time. Model 1 was unadjusted. Model 2 was adjusted for age and sex. Model 3 was fully adjusted for age, BMI, sex, education, race/ethnicity, smoking, alcohol use, hypertension, diabetes, and coronary heart disease (CHD). Continuous HALP was analyzed per 1-standard deviation increase. HALP, hemoglobin-albumin-lymphocyte-platelet score.

**Table S12. Predictive performance of HALP-only models using time-dependent ROC-AUC analysis**

| Cohort / Outcome | Time point | Time-dependent AUC |
| --- | --- | --- |
| WCHAT incidence | 3 years | 0.5377 |
| WCHAT incidence | 4 years | 0.5353 |
| WCHAT incidence | Mean AUC | 0.5365 |
| UK Biobank mortality | 5 years | 0.5739 |
| UK Biobank mortality | 10 years | 0.5835 |
| UK Biobank mortality | 12 years | 0.5524 |
| UK Biobank mortality | Mean AUC | 0.5699 |

Note: Predictive performance was evaluated using HALP-only Cox proportional hazards models and time-dependent receiver operating characteristic curve analysis. In the WCHAT cohort, predictive performance was assessed for incident physical-cognitive comorbidity. In the UK Biobank cohort, predictive performance was assessed for all-cause mortality among participants with baseline physical-cognitive comorbidity. The highest time-dependent AUC was 0.5377 at 3 years for WCHAT incidence and 0.5835 at 10 years for UK Biobank mortality, indicating limited standalone discrimination of HALP. HALP, hemoglobin-albumin-lymphocyte-platelet score; AUC, area under the curve.
